# Introduction to passive electron intensity modulation

**DOI:** 10.1002/acm2.12163

**Published:** 2017-09-06

**Authors:** Kenneth R. Hogstrom, Robert L. Carver, Erin L. Chambers, Kevin Erhart

**Affiliations:** ^1^ Mary Bird Perkins Cancer Center Baton Rouge LA USA; ^2^ Department of Physics and Astronomy Louisiana State University Baton Rouge LA USA; ^3^ _._decimal, LLC Sanford FL USA

**Keywords:** bolus electron conformal therapy, electron beams, intensity modulation

## Abstract

This work introduces a new technology for electron intensity modulation, which uses small area island blocks within the collimating aperture and small area island apertures in the collimating insert. Due to multiple Coulomb scattering, electrons contribute dose under island blocks and lateral to island apertures. By selecting appropriate lateral positions and diameters of a set of island blocks and island apertures, for example, a hexagonal grid with variable diameter circular island blocks, intensity modulated beams can be produced for appropriate air gaps between the intensity modulator (position of collimating insert) and the patient. Such a passive radiotherapy intensity modulator for electrons (PRIME) is analogous to using physical attenuators (metal compensators) for intensity modulated x‐ray therapy (IMXT). For hexagonal spacing, the relationship between block (aperture) separation (*r*) and diameter (*d*) and the local intensity reduction factor (*IRF*) is discussed. The PRIME principle is illustrated using pencil beam calculations for select beam geometries in water with half beams modulated by 70%–95% and for one head and neck field of a patient treated with bolus electron conformal therapy. Proof of principle is further illustrated by showing agreement between measurement and calculation for a prototype PRIME. Potential utilization of PRIME for bolus electron conformal therapy, segmented‐field electron conformal therapy, modulated electron radiation therapy, and variable surface geometries is discussed. Further research and development of technology for the various applications is discussed. In summary, this paper introduces a practical, new technology for electron intensity modulation in the clinic, demonstrates proof of principle, discusses potential clinical applications, and suggests areas of further research and development.

## 1 INTRODUCTION

Intensity modulation (IM) plays an important role in delivering highly conformal, homogeneous (or prescribed heterogeneous) dose distributions tailored to individual patients. Planning and delivery technologies for IM therapies are commercially available for x‐ray therapy using multileaf collimation (MLC) or metal compensators,^1^, ^2^ proton therapy using scanned spot beams,^3^, ^4^ brachytherapy (high‐dose‐rate, computer‐controlled afterloaders and implanted, interstitial source distributions),^5^ and radiosurgery using the gamma knife.^6^ However, intensity modulation for electron therapy is not widely available. This paper will describe a passive radiotherapy intensity modulator for electrons (PRIME) capable of creating IM electron fields.

Three types of electron conformal therapy (ECT) either require or could benefit from intensity modulated fields: segmented‐field ECT, bolus ECT, and modulated electron radiation therapy (MERT).^7^ Heretofore, investigators in these areas envisioned that electron intensity modulation would become available using multileaf collimators (MLCs), similar to x‐ray MLCs used to deliver intensity modulated x‐ray therapy (IMXT). However, the air gap of x‐ray MLCs is too great for their utilization, preventing adequate conformity.^8^ Some envisioned intensity modulation using scanned electron beams,^9^ which requires helium in the treatment head to reduce multiple Coulomb scattering (MCS), as air produces spot beams that are too broad.^10^ This led to work on electron MLC (eMLC) designs by Hogstrom et al.,^11^ Gauer et al.,^12^, ^13^ and others, which resulted in a commercially available eMLC provided by a third party (Euromechanics, Schwarzenbruck, Germany; see http://english.euromechanics.de/electron-multileaf-collimator-emlc/). However, this technology has not resulted in widely available electron IM, possibly due to the high cost of an add‐on eMLC, low fraction of electron patients, lack of its ability to deploy/retract, need for its integration into commercially available treatment planning systems (TPS), and other reasons.

The purpose of the present paper is to introduce the potential for utilization of an alternative method, passive electron intensity modulation, which we believe can be practical for many clinical applications. It is a potentially low‐cost, readily available technology that parallels the use of physical attenuators (metal compensators) for early IMXT,^14^ prior to availability of x‐ray MLCs.^15^ The present paper will discuss the concept of passive intensity modulators, areas of potential clinical applications, various research and development topics to be pursued, and initial work for making the technology clinically available.

## 2 METHODS

### 2.A Concept of passive intensity modulators

The passive radiotherapy intensity modulator for electrons (PRIME) is a device, which when inserted into a therapeutic electron beam, delivers an electron fluence (intensity) distribution that varies (modulates) with position in the plane perpendicular to central beam axis. PRIME consists of a collection of (a) small area island blocks in a plane located inside or just upstream of the aperture of a collimating insert, (b) small area island apertures in a collimating insert, or (c) a combination of both. The locations in the plane and the areas of the island blocks and island apertures are selected to deliver a desired intensity‐modulated electron fluence distribution.

**Figure 1:**
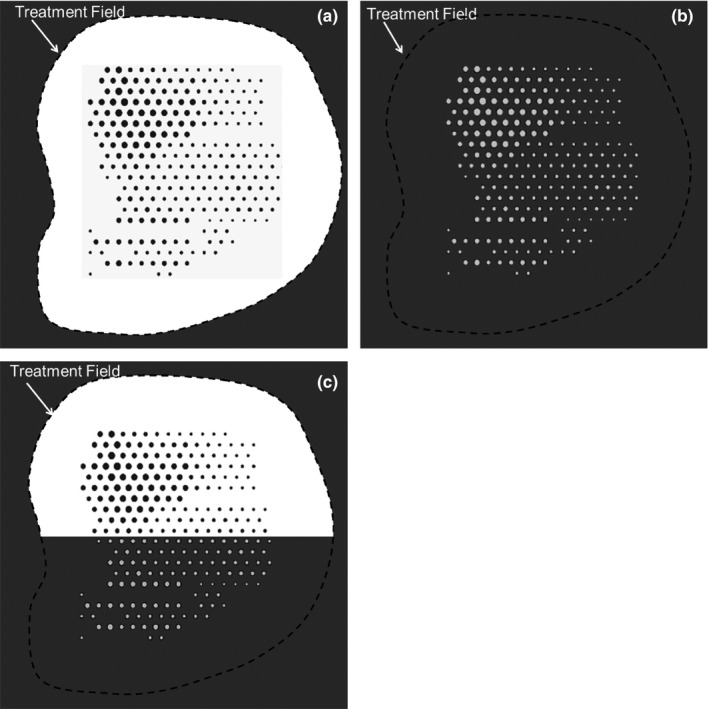
Illustration of types of PRIME devices located inside or just upstream of electron collimating insert. (a) island blocks (dark gray circles) inside collimating aperture (black dashed curve) for 50% ≤ *I(x,y) *≤ 100%; (b) island apertures (light gray circles) inside virtual collimating aperture (black dashed curve) for 0% ≤ *I(x,y) *≤ 50%; (c) Island blocks (dark gray circles) and island apertures (light gray circles) inside electron collimating aperture. Treatment field (black dashed curve) is composed of both actual aperture with island blocks and virtual aperture in collimating insert with island apertures. *I(x,y)* is the modulated relative electron intensity at off‐axis position *(x,y)*.

As an example, Fig. 1(a) illustrates a collection of circular island blocks of varying diameter located on a hexagonal grid inside the aperture of a custom electron collimating insert. Figure 1(b) illustrates a collection of island apertures of varying diameters located on a hexagonal grid inside the virtual aperture of a custom electron collimating insert. Figure 1(c) illustrates a combination of the two. The island blocks and island apertures, shown located on a hexagonal grid in Fig. 1, can be located in any spatial pattern that achieves the desired intensity pattern. Their cross‐sectional area can be circular, square, hexagonal, or other shape, although circular shape has the smallest ratio of side surface area to incident area, which should minimize the undesirable effect of MCS electrons escaping the collimating material of the island blocks or island apertures. The thickness of the high‐density island block and the thickness of the collimating insert containing the island apertures should be sufficient to stop primary electrons.

Never before has multiple island blocks or island apertures been used to provide electron intensity modulation. Previously, single circular island blocks have been used to protect the lens of the eye, located approximately 0.7 cm under the eye surface, while treating the underlying retina to approximately 70% of “given” dose.^16^, ^17^ Also, saw‐toothed collimator edges have been used to broaden the penumbra (80%–20%) to match the penumbra of abutting electron fields of differing energies.^18^ Although both applications are similarly based on principles of MCS, neither used multiple island blocks to generate an electron beam with full field intensity modulation.

#### 2.A.1 Intensity modulation 50%–100%

**Figure 2:**
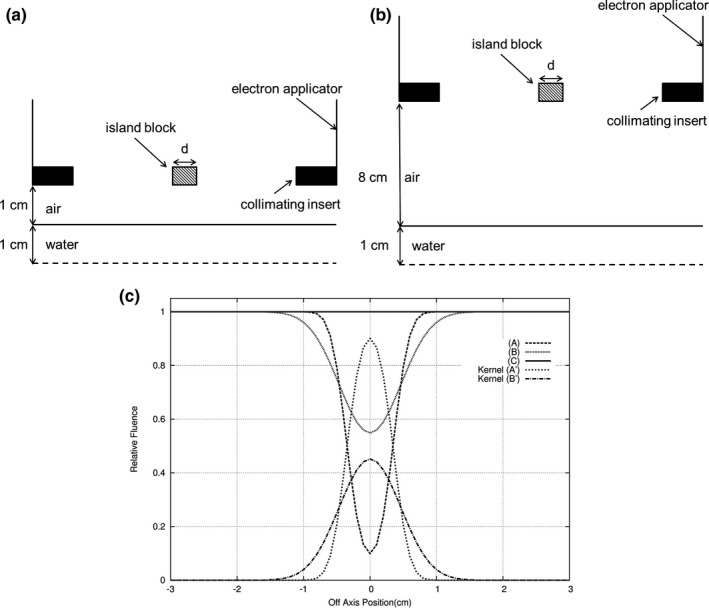
Illustration of electron fluence profiles under 0.7‐cm diameter island block for 16 MeV beam, 15 × 15‐cm^2^ field. Cross‐section of measurement geometries (not to scale) for (a) profile A (air gap = 1 cm; depth in water = 1 cm) and (b) profile B (air gap = 8 cm; depth in water = 1 cm). (c) Comparison of profiles (relative fluence versus off‐axis position) for no island block (C), island block with 1‐cm air gap (A), and island block with 8‐cm air gap (B). Subtracting profile A from C and B from C result in fluence kernels (fluence removed from the beam) A’ and B’, respectively.

The island block removes most, ideally all, electrons incident on its entry surface from the beam. Figure 2 illustrates for a parallel beam the relative electron fluence (intensity) for the central region of the beam (a) 1‐cm depth in water with 1‐cm air gap under the island block, (b) 1‐cm depth in water with 8‐cm air gap under the island block, and (c) without the island block. As the distance between the island blocks and the plane of calculation increases, the resulting electron fluence distribution broadens due to (a) MCS of electrons in air upstream of the IM plane creating an initial angular distribution at each point in the IM plane and (b) MCS of electrons downstream of the IM plane by the water phantom (or patient). Also shown is the difference between the fluence distributions (without island block and with island block). This difference is the kernel representing the electron fluence removed from the beam by a single island block.

**Figure 3:**
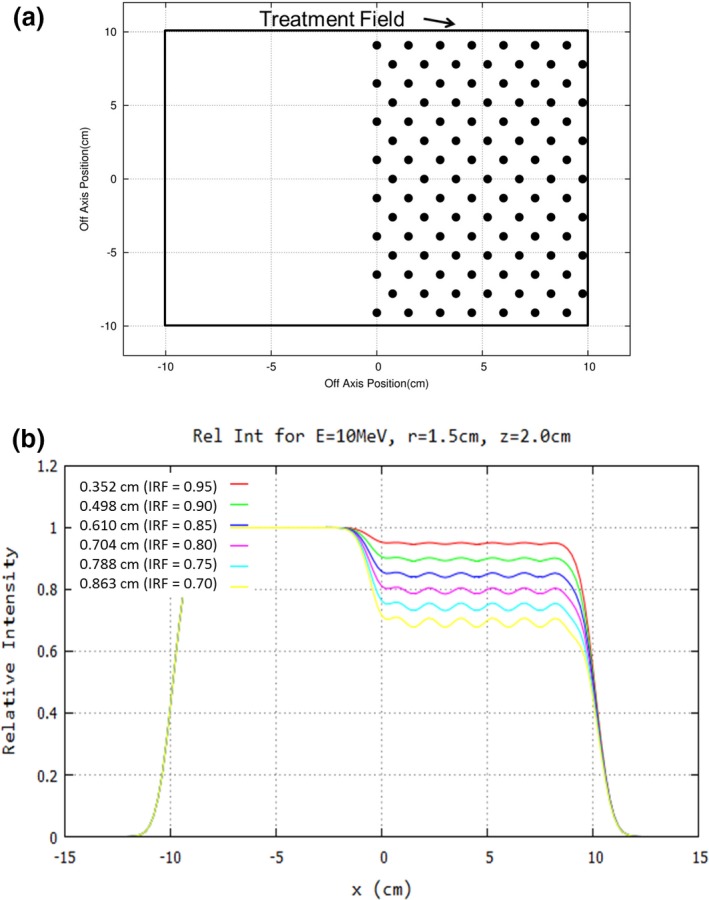
Concept of intensity modulation illustrated for half‐field (20 × 20 cm^2^) at 10 MeV. (a) Beams‐eye‐view (BEV) shows island blocks (black circles, diameter d = 0.5 cm) packed in a hexagonal matrix (separation r = 1.5 cm) resulting in an average intensity reduction to 90% of intensity without island blocks. (b) y = 0, x‐profiles at 2‐cm water depth for 8‐cm air gap show the relative fluence for reduced intensities of 95%, 90%, 85%, 80%, 75%, and 70% (d = 0.352, 0.498, 0.610, 0.704, 0.788, and 0.863 cm, respectively). Note that magnitude of ripples increases (decreases) with increasing (decreasing) d.

In Fig. 3, a half field of 0.5‐cm diameter island blocks separated by 1.5 cm is shown, along with the underlying intensity (relative fluence) distributions (i.e., intensity distribution without island blocks less the composite of the kernel distributions) for different diameter blocks. The resulting y = 0 profiles show a decreased intensity in the +x domain, where away from the edge (x = 0) the average reduction in intensity equals the fraction of the beam blocked by the island blocks. By properly selecting the area and the separation of the island blocks in the locally blocked area, the desired transmitted intensity can be closely achieved locally. For example, for hexagonally packed circular island blocks of diameter (d) and separation (r), the reduced local intensity is given by

(1) $$I_{island\;blocks}\left(d,r\right)=I_{o}\left[1-\left(\frac{\pi }{2\sqrt{3}}\right){\left(\frac{d}{r}\right)}^{2}\right]\;,$$

where $I_{o}$ is the intensity with no island blocks. This formula allows an estimate of the block diameter at each point on a hexagonal grid to be calculated based on the desired underlying intensity,

(2) $$d\left(r,IRF\right)=r{\left[\frac{2\sqrt{3}}{\pi }\left(1-IRF\right)\right]}^{1/2},$$

where *IRF* = *I*_*desired*_/*I*_0_ is the desired underlying intensity reduction factor. Because each island block impacts multiple locally desired intensities due to MCS of the electrons, an optimizer based on an inverse planning algorithm is required to determine an optimal intensity modulator design. This should be a function for the treatment planning system.

#### 2.A.2. Intensity modulation 0%–50%

In this case, more than half of a local area of the field is blocked, for which the same principles as above apply, but for small island apertures in the collimating insert, as opposed to small island blocks within the aperture of the collimating insert.

In the central region of the beam, the relative electron fluence (intensity) in water (1‐cm depth) located 1 cm and 8 cm behind a single, small island aperture equals the subtraction kernels shown in Fig. 2; without the island block aperture no electron intensity is transmitted. Again, as the distance between the island apertures and the plane of calculation increases, the resulting electron fluence distribution broadens due to MCS of electrons, as discussed earlier.

**Figure 4:**
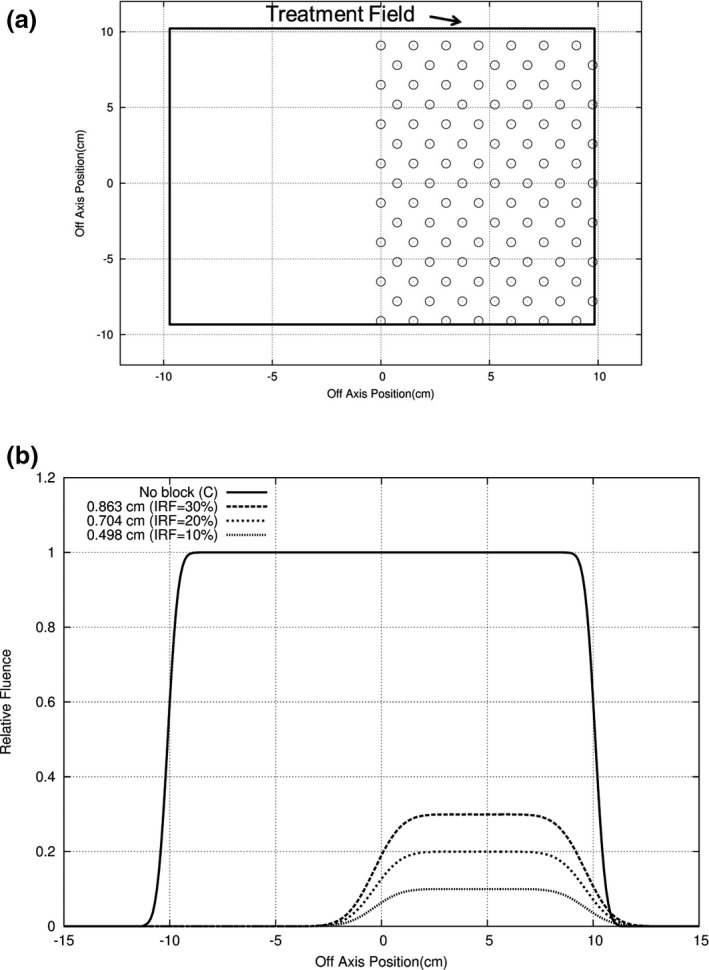
Concept of intensity modulation illustrated for half‐field (20 × 20 cm^2^) at 10 MeV. (a) Beams‐eye‐view (BEV) shows island apertures (open circles, diameter d = 0.5 cm) packed in a hexagonal matrix (separation r = 1.5 cm) resulting in an average intensity transmission of 10% of intensity for open 20 × 20 cm^2^ field. (b) y = 0, x‐profiles at 2‐cm water depth for 8‐cm air gap show the relative fluence for reduced intensities of 30%, 20%, and 10% (d = 0.863, 0.704, and 0.498 cm, respectively).

In Fig. 4, a half beam of island apertures (0.5 cm diameter) located on a hexagonal grid and separated by 1.5 cm is shown. The resulting profiles shows an increasing intensity at the edge (x = 0), and away from the edge, the intensity equals the fraction of the beam unblocked by the island apertures (10, 20, and 30%). By properly selecting the size and the separation of the island apertures within the local area, the desired intensity can be achieved locally. For example, for hexagonally packed circular island apertures of diameter d and separation r, the local intensity is given by

(3) $$I_{island\;apertures}=I_{0}\left(\frac{\pi }{2\sqrt{3}}\right){\left(\frac{d}{r}\right)}^{2}.$$

The formula allows an estimate of the aperture diameter at each point on a hexagonal grid to be calculated based on the desired underlying intensity,

(4) $$d\left(r,IRF\right)=r{\left[\frac{2\sqrt{3}}{\pi }IRF\right]}^{1/2}.$$

Because each island aperture impacts multiple locally desired intensities due to MCS of the electrons, an optimizer based on an inverse planning algorithm is required to determine an optimal intensity modulator design, and again this should be a function for the treatment planning system. Furthermore, since the primary application of island apertures for producing intensities in the range 0%–50% might be for MERT, for which intensities vary 0%–100%, island blocks would likely be used in conjunction with island apertures.

### 2.B Range of island block parameters (d,r) for range of intensity reduction factors

Knowledge of the useful range of sizes (cross‐sectional area) for island blocks and island apertures is desirable. Island blocks of circular cross‐section packed in a hexagonal grid should be useful for intensity modulated bolus ECT, where IRFs in the range of 0.70 to 1.00 are expected.^19^ As IRF depends on (d/r) in Eqs. (1) and (1), multiple solutions of (d,r) can provide a desired IRF (0.70–1.00). The larger the values of (d,r), the fewer the number of island blocks required and the smaller the effect of electron scattering off the blocks, both advantageous and the latter due to reducing the surface area of the block edges. However, making values of (d,r) too large does not allow sufficient scatter beneath the blocks to create a uniform, reduced fluence (intensity). We consider (d,r) values acceptable if intensity is within 2% of the desired IRF.

This effect has been studied for multiple electron energies (7–20 MeV) at two SSDs (100 and 103 cm SSD) and water depths of 0.5 and 2.0 cm.^20^ Calculations using the Hogstrom et al. pencil beam algorithm (PBA),^21^ which assumed perfect collimation, were used to determine acceptable (d,r) values for achieving clinical intensity reduction factors (0.70 ≤ IRF ≤ 0.95). Intensity distributions were calculated beneath a half‐blocked 20 × 20 cm^2^ field (*cf*. Fig. 3) for 0.5 ≤ r ≤ 1.5 cm and 0.70 ≤ IRF ≤ 0.95, for which 0.117 ≤ d ≤ 0.863 cm. Samplings of the results are reported here with a broader, comprehensive range to be reported in a subsequent manuscript.

### 2.C Construction of intensity modulators

Methods for the construction of intensity modulators are presently under development. This section discusses construction specifications and a prototype, fabricated to illustrate proof of principle.

#### 2.C.1 Construction constraints

Optimally, the intensity modulator will be small cross‐section island blocks and island apertures strategically located in the collimating space occupied also by the custom electron insert. Island blocks could be cylinders fixed in space in the aperture of the insert collimator, achieved by embedding them in a low‐density foam that could be accounted for in the fluence calculation, but have little impact. Island apertures could be circular holes in the electron insert's collimating portion. Ideally, the central axes of both the island blocks and island apertures should follow the diverging rays emanating from the virtual source of the electron beam. Also, the shape of the cross‐section of the island blocks and island apertures should be circular, forming right oblique cylinders whose axes coincide with diverging ray lines. These two properties should minimize the effect of electrons scattered from the walls of the island blocks and island apertures.

The island apertures will be the same thickness (g cm^−2^) as the custom electron insert, usually sufficient to stop electrons from the highest beam energy (20 MeV). It is recommended that the island blocks be the same thickness so as to be able to be used at all electron energies. The collimating material should be a high density metal, possibly the same or similar material as the custom electron inserts. Presently, most inserts are fabricated using low melting point lead alloy (Cerrrobend)^22^ or copper.^23^ Tungsten alloy is another potential material for the island blocks, its being denser, harder, and less toxic than lead, all advantages for a block material.

#### 2.C.2 Island block intensity modulator proof of principle

Initial proof of principle compared measurement with calculation for a prototype IM. The prototype was constructed by inserting lead wire (0.2‐cm diameter × 2.0‐cm thick) into a 2.0‐cm thick piece of Styrofoam on a hexagonal grid with r = 0.5 cm, corresponding to an IRF of 0.85. The block matrix, which consisted of five rows of 6–7 pins with the central pin located at central axis, was abutted to the upstream side of the final trimmer. Relative dose measurements were made along x = 0 (in‐plane) with a 16 MeV electron beam and 10 × 10‐cm^2^ field at 2.0‐cm depth (100‐cm SSD) on an Elekta Infinity accelerator. These measurements were made using a p‐type electron dosimetry diode detector (EFD^3G^, #300–605) with an active volume diameter of 0.2 cm and thickness of 0.006 cm (IBA Dosimetry, Bartlett, TN, USA). The diode was connected to the scanning main control unit of the RFA‐200 Water Phantom 2D scanning tank using OmniPro scanning software (IBA Dosimetry, Bartlett, TN, USA). For comparison, the off‐axis dose profile was also calculated using the PBA for identical conditions.

#### 2.C.3 Patient example

Kudchadker et al.^19^ showed how IM improved planning target volume (PTV) dose homogeneity for bolus ECT of a head and neck patient (right buccal mucosa). We used the reported intensity distribution for that patient to design an intensity modulator, which closely provided the desired IM dose distribution at a depth of 2 cm in water. Details of the design process for the intensity modulator^20^ remain under investigation and will be reported later, but preliminary results for this patient are shown.

## 3 RESULTS

### 3.A Range of island block parameters (d,r) for range of intensity reduction factors

**Figure 5:**
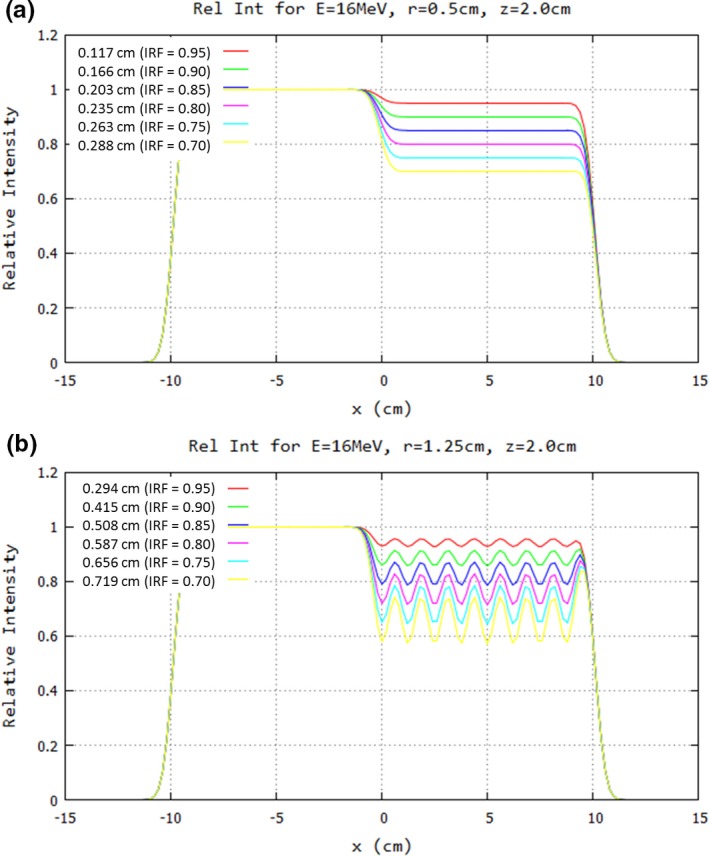
Impact of hexagonal spacing on profiles under half‐field of island blocks of 20 × 20 cm^2^ field at 16 MeV. Profiles at 2‐cm depth in water (103‐cm SSD) are plotted for IRF values of 0.70–0.95 and hexagonal spacing of (a) r = 0.5 cm and (b) r = 1.25 cm. Note that magnitude of ripples increases (decreases) with increasing (decreasing) r, and hence d, at the (d/r) value for a specified IRF. Key shows values for d preceding each IRF value.

Although a detailed compilation of extensive (d,r) results will be the subject of a separate manuscript, Fig. 5 illustrates results at 16 MeV, 103‐cm SSD, and 2.0‐cm depth, for r = 0.5 and 1.25 cm. Results at r = 0.5 cm for IRF = 0.70 to 0.95 (d = 0.117 to 0.288 cm) show smooth profiles with constant intensity reduction equaling the IRF under the island blocks predicted using Eq. (1); results at r = 1.25 cm for IRF = 0.70 to 0.95 (d = 0.294 to 0.719 cm) show oscillations about the desired IRF by approximately ± 1.5% for an IRF of 0.95 and ± 7.5% for an IRF of 0.70, the latter being unacceptable.

**Figure 6:**
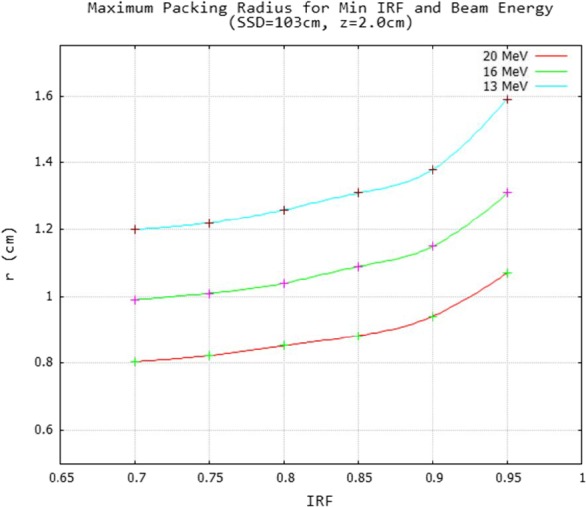
Energy dependence of maximum hexagonal packing radius for minimum IRF of an intensity modulator. For the minimal IRF value (0.7–0.95) the maximum hexagonal packing is plotted for which ripples determined from half‐field modulated fields equaled ± 2% for 13, 16, and 20 MeV beams. A smooth curve was drawn through the data points calculated at 0.05 increments of IRF for a 2.0 cm water depth (103 cm SSD).

Figure 6 shows data which allow determination of the maximum packing radius values for the minimal IRF value (0.70 to 0.95) in an IM field, yet still being able to achieve profile values within 2% of desired IRF values. These data were for 13, 16, and 20 MeV beams, SSD = 100 cm, and 2‐cm depth in water. At 20 MeV, r ranges from approximately 0.8–1.07 cm, which corresponds to d values of approximately 0.46–0.25 cm; at 16 MeV, r ranges from approximately 1.0–1.3 cm, which corresponds to d values of approximately 0.58–0.31 cm; and at 13 MeV, r ranges from approximately 1.2–1.6 cm, which corresponds to d values of approximately 0.69–0.38 cm. These values change with SSD and depth, which depends on the bolus shape and position.

### 3.B Island block intensity modulator proof of principle

**Figure 7:**
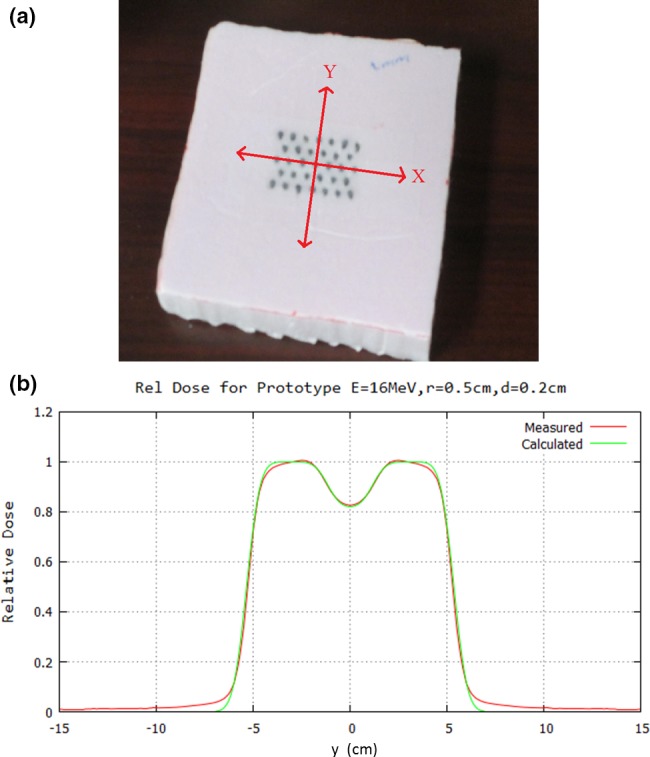
Comparison of PBA‐calculated with measured relative dose profile under prototype intensity modulator (IM). (a) Bird's‐eye‐view of prototype IM consisting of 5 rows of 6 or 7 island blocks (0.2‐cm diameter lead wire, 2‐cm long snippets) inserted into Styrofoam block on hexagonal grid (r = 0.5 cm). The IM was placed on top of the electron insert. (b) Dose is compared 7 cm downstream of the 10 × 10 cm^2^ field (2 cm depth in water at 100‐cm SSD). PBA calculated dose (electron component only) is compared with that measured (electron and x‐ray component) using a scanning diode. Note Eq. (1), using the (d/r) value of 0.4, yields IRF = 0.85, close to the measured and calculated minimum of 0.82.

Results of the measurement and PBA calculation for a prototype intensity modulator constructed of Styrofoam and lead wire [*cf*. Fig. 7(a)] are compared in Fig. 7(b). Off‐axis ratios (OARs) showed approximately 1% agreement within the modulated area. The minimum relative dose in the field was 0.82, close to the 0.85 value calculated using Eq. (1).

### 3.C Example design of intensity modulator for buccal mucosa patient

**Figure 8:**
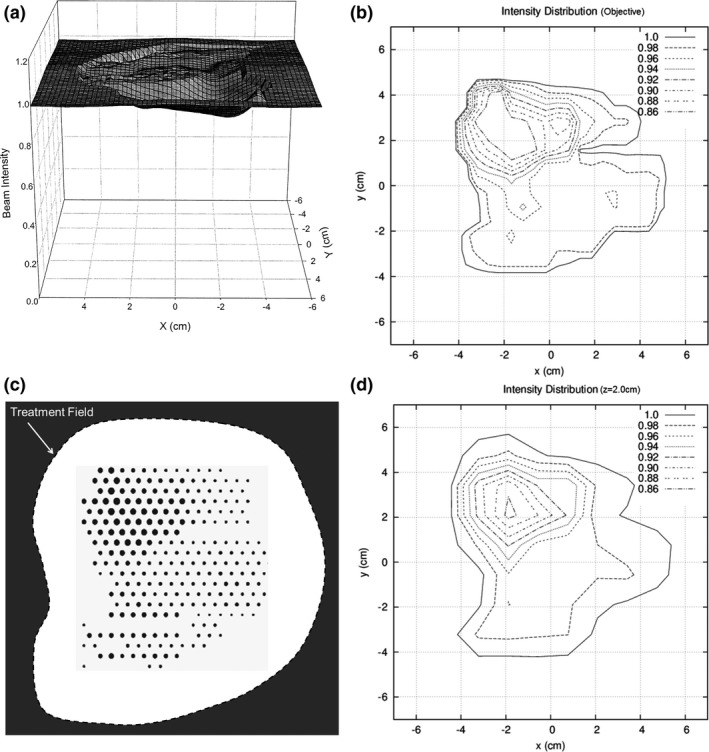
Illustration of intensity modulator designed for bolus ECT patient. (a) Plot of intensity modulation distribution determined to improve PTV dose homogeneity for buccal mucosa bolus ECT dose plan (from Kudchadker et al.^19^). (b) Isocontour plot of intensity modulation distribution manually constructed from plot in (a). (c) Beams eye view of island block matrix on a hexagonal grid (r = 0.5 cm), which consists of variable diameter cylindrical blocks (d ≤ 0.26 cm for IRF ≥ 0.70) selected to deliver reconstructed isointensity pattern for a 20‐MeV electron beam at 2‐cm depth in water (103‐cm SSD). (d) PBA‐calculated isointensity pattern produced by the island block matrix for a 20‐MeV electron beam at 2‐cm depth in water (103‐cm SSD).

Figure 8 illustrates how an intensity modulator might be designed to deliver a desired intensity pattern for IM bolus ECT. Figure 8(a) shows the desired intensity modulation to improve PTV dose homogeneity for a buccal mucosal cancer treated with bolus ECT.^19^ Figure 8(c) shows a BEV of a set of island blocks on a hexagonal grid (r = 0.5 cm) for which the diameters have been optimized using an algorithm based on Eq. (1).^20^ Iso‐intensity plots of the desired intensity pattern constructed from Fig. 8(a) and then computed at a 2‐cm depth in water (103 cm SSD) using a PBA are compared in Figs. 8(b) and 8(d), respectively. In other words, the intensity modulation provided by the island blocks at the plane of collimation results in the desired intensity modulation pattern at depth. Similar results were achieved at 0.5 cm depth.^20^

## 4 DISCUSSION

### 4.A Areas of potential clinical applications

#### 4.A.1 Intensity modulated bolus electron conformal therapy (ECT)

Bolus electron conformal therapy^24^ is the only ECT technology for which planning and delivery tools presently are commercially available. (.decimal, LLC, Sanford, FL, USA, http://dotdecimal.com/products/electrons/bolusect/). Clinical sites for which bolus ECT is useful are well documented in the literature, for example, posterior wall sarcomas,^25^ postmastectomy chest wall,^26^, ^27^, ^28^ head and neck,^29^, ^30^ and extremities,^31^ and on the.decimal web site.

One area of improvement for bolus ECT is that the bolus, which rests on the patient surface, has an irregular upstream surface designed to conform the therapeutic dose surface (e.g., 90%) to the distal PTV surface. This irregular surface produces hot/cold spots, increasing dose spread in the PTV from 10% (90%–100%) to as much as 30%. Kudchadker et al.^19^ showed that IM bolus ECT can restore the dose spread to 10%–12%. Preliminary investigation by Chambers^20^ showed how IM composed of variable‐diameter island blocks spaced on a hexagonal grid can provide the needed electron beam intensity modulation.

#### 4.A.2 Segmented‐field electron conformal therapy

Segmented‐field electron conformal therapy utilizes multiple (two or more) electron fields of differing energy abutted (patched) together.^7^, ^32^ However, abutted electron fields of differing energies have differing penumbra widths, creating dose heterogeneity (hot/cold spots) in the abutting region. This dose heterogeneity can be reduced by matching penumbras of abutted fields, which is possible by increasing the sharper penumbra of the higher energy field to match that of the lower energy field. This can be done by placing higher energy field collimating apertures farther from the patient^33^ or by modulating field edges using an eMLC^34^; however, neither of these technologies is commercially available. Passive intensity modulators with island blocks and island apertures located near the edges of a field could provide this function, analogous to that previously reported using saw‐toothed collimator edges.^18^

#### 4.A.3 Modulated electron radiation therapy (MERT)

MERT utilizes multiple intensity modulated electron fields of varying angle and energy.^35^, ^36^, ^37^, ^38^, ^39^ Ma et al. reports MERT being delivered using an eMLC^39^; however, PRIME might be able to be used for MERT. Because MERT requires a full range of modulation (0%–100%), its use of the passive modulators would require both island blocks and island apertures, creating the greatest challenge. Clinical and technical issues, which could make passive intensity modulation less desirable for MERT, include (a) the inefficiency of multiple room entries to change the IM for each of the multiple electron fields, (b) the impact of scattering from the walls of the island blocks and apertures, and (c) increased bremsstrahlung dose penetrating the collimating metal, the latter two arising from the increased number of monitor units possibly required to deliver a MERT plan.

#### 4.A.4 Variable surface

It is well known that irregular variations in the patient surface can create hot/cold spots due to MCS and that angled incidence can increase and/or decrease dose due to variations in SSD.^40^ It should be possible to reduce both effects by using passive intensity modulators. Examples might be the nose, limbs, and postmastectomy chest wall.

### 4.B Potential future research and development

Planning and delivery tools necessary for clinical uses of PRIME provide multiple opportunities for research and development. These include, but are not limited to:

1. Optimization algorithms (inverse planning) and software for determining an acceptable set of island blocks and island apertures (defined by their cross‐sectional areas and positions) that provide a desired intensity pattern and underlying patient dose distribution;
2. Dose calculation engines that can calculate absolute dose (dose per MU) distributions resulting from PRIME with speed, accuracy, and utility sufficient for clinical use;
3. Documentation of accuracy of dose calculations using measured data; and
4. Methods for fabrication of intensity modulators.

Clinical utilization of these tools provides opportunities for clinical research and development. These include, but are not limited to:

- Methods for incorporating intensity modulation into the treatment planning process, for example, for IM bolus ECT, modulation could become another operator in the bolus design process^24^, ^31^;
- Treatment planning protocols for incorporating the technology into current clinical practice;
- Assessment of utility of IM through treatment planning studies comparing IM electron plans with other modalities for specific sites; and
- Methods for quality assurance of electron intensity modulators.

### 4.C Making the technology clinically available

Widespread access to IM electron beam technology for clinical use requires commercial availability of treatment planning and delivery technology. To date, only bolus electron conformal therapy, one of three types of electron conformal therapy,^7^ is commercially available. That product, BolusECT® software in the p.d planning system (.decimal, LLC, Sanford, FL, USA), designs an electron bolus and returns it to the host treatment planning system via DICOM for final dose calculation and approval. However, current commercial electron beam planning systems cannot perform IM electron dose calculations, an issue which must be resolved in either p.d or radiotherapy treatment planning systems. Regarding development of a commercial system, the authors recently received a Phase I Small Business Technology Transfer (STTR) grant from the National Institutes of Health (NIH) to investigate and develop a prototype IM bolus ECT planning and delivery system, which includes preliminary research and development for some of the items listed in the previous section.

## 5 SUMMARY

This paper introduces for the first time a new, potentially practical method for using inexpensive, passive intensity modulators for intensity modulated electron therapy. The passive radiotherapy intensity modulator for electrons (PRIME) consists of a set of island blocks and island apertures appropriately sized and spaced to create the desired intensity pattern. PRIME functions on the premise that diameter and spacing of island blocks and island apertures can be selected so that MCS fills in dose behind island blocks and between island apertures such that locally there is an intensity reduction related to the fraction of beam blocked.

Applications of intensity modulators for multiple forms of modulated electron therapy^7^ were discussed. The present authors are focusing on its application to bolus ECT, an existing clinically available technology. The relationship between block diameter and separation of cylindrical blocks on a hexagonal grid was illustrated for bolus ECT, and subsequent publications will report more details of these results. Proof of principle was illustrated by comparing PBA calculated with measured dose for a prototype IM and by designing an IM for a head and neck patient previously treated with bolus ECT.

## CONFLICTS OF INTEREST

Kevin Erhart is an employee of.decimal, LLC and principal investigator of NIH Award Number R41CA199838 for which Mary Bird Perkins Cancer Center has a consortium agreement. The authors have no other relevant conflicts of interest to disclose.
